# Increasing the Response of Mismatch Repair Proficient Rectal Cancer to Immunotherapy with Particle Radiation and DNA Damage Response Inhibitors—Preclinical Evidence

**DOI:** 10.3390/cancers18040682

**Published:** 2026-02-19

**Authors:** Cristian J. Salazar-Vilches, Daniel K. Ebner, Jake A. Kloeber, Sonja Dragojevic, Jasvinder Singh, Michael Haddock, Yasamin Sharifzadeh, Alexander D. Sherry, Krishan R. Jethwa, Christopher L. Hallemeier, Kenneth W. Merrell, Robert W. Mutter, Zhenkun Lou, Cameron M. Callaghan

**Affiliations:** 1 Department of Radiation Oncology, Mayo Clinic, Rochester, MN 55905, USA; salazarvilches.cristian@mayo.edu (C.J.S.-V.);; 2 Department of Oncology, Mayo Clinic, Rochester, MN 55905, USA

**Keywords:** rectal cancer, particle therapy, immunotherapy, DNA damage response inhibitors

## Abstract

Background/Objectives: We performed a systematic review of preclinical literature on the use of high-LET particle therapy, DDRi, and/or immunotherapy specifically in pMMR colorectal cancer. Methods: A systematic review of the literature published between 2014 and 2025 was conducted across major databases. Studies were included if they examined particle radiotherapy (e.g., proton, alpha, and carbon) or X-ray radiation either alone or in combination with DDRi and/or immune checkpoint inhibitors (ICIs) in pMMR colorectal cancer models. Results: In total, 131 studies met the inclusion criteria, including 70 preclinical studies. These studies consistently demonstrate that high-LET radiation amplifies immunogenic cell death, increases cGAS-STING pathway activation, and enhances tumor antigen presentation, thereby fostering greater immune infiltration and systemic antitumor responses. Concurrent irradiation with DDRi enhances persistent DNA damage and cytosolic DNA accumulation. In murine models, high-LET therapies show excellent local control, with manageable toxicity profiles. Combination regimens with ICIs exhibit improved local control and elicit systemic antitumor immune responses. Conclusions: High-LET particle radiation and/or the use of concurrent DDRi with ICI have significant preclinical evidence of immunostimulatory effects in pMMR rectal adenocarcinoma and increased response rates to immunotherapy. The clinical evidence will be reviewed in the companion manuscript.

## Simple Summary

Patients with mismatch repair deficient (dMMR) rectal adenocarcinomas demonstrate significant complete response rates to PD-1 inhibitor monotherapy, potentially allowing them to avoid chemotherapy, radiation, and surgery. However, >95% of patients are mismatch repair proficient (pMMR) and have so far not responded to immunotherapy in clinical trials. There is growing evidence that both high linear energy transfer (LET) particle radiation and DNA damage response inhibitors (DDRis) may increase the response of pMMR rectal cancer to immunotherapy and are ready for clinical translation.

## 1. Introduction

Colorectal cancer is a leading cause of cancer-related mortality, with an increasing incidence among younger patients [1,2]. While mismatch repair deficiency (dMMR) confers a substantial mutational burden and robust response to immunotherapy, over 95% of rectal cancer patients are MMR proficient (pMMR) [3] and do not respond to immunotherapy alone [4].

Standard treatment for locally advanced pMMR rectal adenocarcinoma includes ionizing radiation (IR), chemotherapy, and surgery or watch-and-wait surveillance for patients with a complete clinical response after total neoadjuvant therapy (TNT) [5]. TNT improves organ preservation and disease outcomes. However, with standard TNT, only ~20–40% of patients achieve a complete clinical response, and those opting for nonoperative management (NOM) remain at risk of local and distant recurrence. Thus, adding immunotherapy to TNT to improve the response in pMMR disease could improve outcomes, reduce toxicity, and increase the proportion of patients eligible for NOM [6,7].

Immune checkpoint inhibitors (ICIs), most commonly targeting PD-1/PD-L1 and/or CTLA-4, aim to restore antitumor T-cell activity by blocking inhibitory immune signaling. In colorectal cancer, complete and durable responses to checkpoint blockade monotherapy are largely confined to dMMR tumors, whereas the majority of patients with pMMR disease do not respond. This unmet need motivates combination approaches that increase tumor immunogenicity and immune infiltration [8].

One strategy to increase the response of pMMR disease to ICI is by targeting DNA damage response (DDR) pathways (either by selecting patients with intrinsic defects/somatic mutations or via pharmacologic inhibition). Ineffectual MMR and homologous recombination (HR) are most frequently found to increase tumor mutation burden, genomic instability, and neoantigens and potentially sensitize tumors to ICI [9,10,11]. This has been demonstrated most dramatically by the complete response rates of dMMR rectal cancers to PD-1 inhibition [12]. Mutations leading to DDR defects may also predict synthetic lethality with DDR inhibitors (DDRis), as seen in BRCA mutants and PARP inhibitors. Inactivation of DDR pathways (via loss-of-function mutations or pharmacologic inhibition) can further enhance immune-based therapies by amplifying DNA damage signaling and antitumor immune responses [9,13,14]. Because IR is a clinically established way to induce DNA damage, the quality of the damage may be critical for synergy between DDRis and ICIs.

One aspect of IR known to contribute to the quality/complexity of DNA damage is the linear energy transfer (LET) of radiation, with conventional X-rays (XRT) having low-LET and alpha/carbon/iron particle beams exhibiting high-LET. LET describes how densely radiation deposits energy along its track through the tissue (LET = dE/dℓ; typical units: keV/µm). Compared with low-LET XRT, high-LET radiation produces more complex clustered damage around double-strand breaks (DSBs). Repair of these complex DSBs often relies on error-prone repair pathways, promoting genomic instability and generating potent immunogenic stress signals [15,16,17]. Concordantly, the addition of DDRi to high-LET further enhances radiosensitization compared to XRT [17,18,19] and may elicit a more robust immune response [20].

In this review, we systematically examine the preclinical evidence on optimal combination therapies using radiation, DDRi, and ICI for local and systemic control in pMMR CRC, with a focus on the subclass of IR/DDRi/ICI, dosage/scheduling, and potential mechanisms of synergy or immune priming. We highlight clinically actionable variables, LET, dose/fractionation, and sequencing to inform future translation of combination therapy regimens into early-phase clinical trials.

## 2. Materials and Methods

### 2.1. Literature Search

This search aimed to identify preclinical studies focusing on particle radiation (especially with direct comparisons to XRT) alone or in combination with ICI and/or DDRi. XRT studies were included when they served as comparators for particle radiation or when in combination regimens with DDRi/ICI in pMMR colorectal cancer models. Synthesis and review of clinical studies are reported in the companion manuscript. We searched PubMed Central, Cochrane Library, Scopus, Epistemonikos, Web of Science, and Google Scholar for articles published between 1 January 2014 and 31 August 2025 (date of last search) using combinations of terms related to rectal cancer and high-LET radiation therapies. Search terms included variations of “rectal cancer,” “colorectal cancer,” “rectal carcinoma,” “colorectal carcinoma,” “rectal tumor,” and “colorectal tumor,” combined with terms such as “high LET radiation,” “high linear energy transfer radiation,” “proton therapy,” “carbon ion radiation therapy,” “CIRT,” and “diffusing alpha-emitter radiation therapy” or “DaRT.” For studies examining combination therapies, additional terms included “immunotherapy,” “PD-1,” “PD-L1,” “CTLA4,” “immune checkpoint inhibitors,” “DNA damage response inhibitors,” “PARP inhibitors,” “ATR inhibitors,” “ATM inhibitors,” “DNA-PK inhibitors,” “WEE1 inhibitors,” “CHK1 inhibitors,” and “CHK2 inhibitors” (including “inhibition” instead of “inhibitors”). The search strategies were adapted for each database. The PICO search strategy, specific search strategies, and keywords for pMMR status identification are presented in Supplementary Materials.

**Figure 1:**
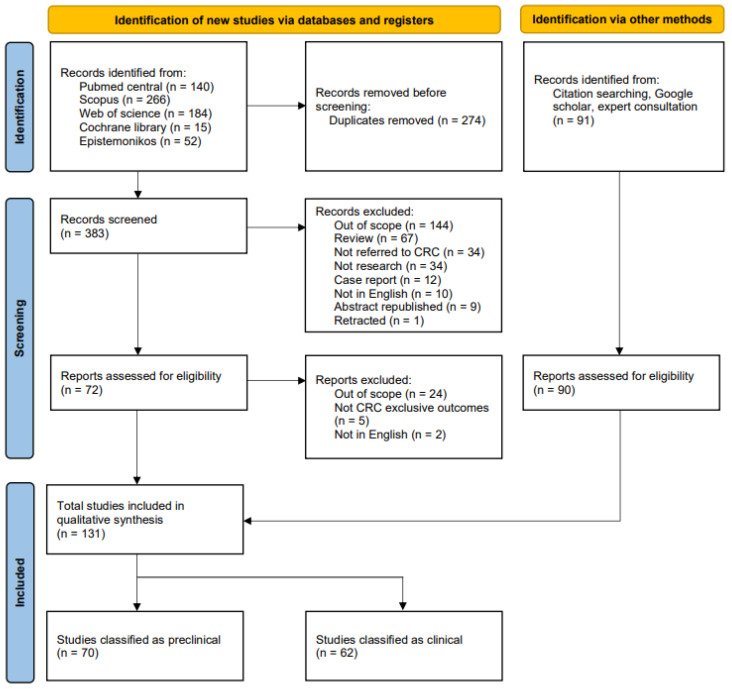
PROSPERO flowchart for systematic literature search for both clinical (current manuscript) and preclinical data (companion manuscript). The collection, inclusion, and exclusion process of the systematic literature search adhered to PROSPERO guidelines (registration #CRD42024627912 at the PROSPERO website (https://www.crd.york.ac.uk/PROSPEROFILES/627912_STRATEGY_20250208.pdf). Specific search criteria are detailed in Supplementary Materials.

References cited in the included studies were manually reviewed to identify additional relevant articles. Two independent investigators (C.C. and C.S.) screened titles, abstracts, and full-text articles for inclusion. Studies were eligible if they investigated particle radiation, either as monotherapy or in combination with ICI or DDRi, in preclinical colorectal cancer. The search was limited to articles published in English. Discrepancies were resolved through consensus. This systematic review adhered to the Preferred Reporting Items for Systematic Reviews and Meta-Analyses (PRISMA; Figure 1) guidelines [21]. The review search protocol can be accessed at (https://www.crd.york.ac.uk/PROSPEROFILES/627912_STRATEGY_20250208.pdf) or review registration # CRD42024627912 on the PROSPERO website.

### 2.2. Review and Synthesis

Preclinical studies were categorized based on the treatment modality: XRT, proton beam therapy (PBT), carbon-ion radiation therapy (CIRT), and alpha irradiation or diffusing alpha-emitter radiation therapy (DaRT), alone or in combination with ICI and/or DDRi. Due to the heterogeneity of the interventions and endpoints reported in the various studies, meaningful quantitative synthesis was not possible, so topical qualitative synthesis was conducted. Similarly, the risk of bias was generally similar to that of preclinical studies; however, pertinent limitations regarding the interpretation of study results are discussed throughout. Comparisons with conventional XRT vs. particle radiation or between classes of ICI or DDRi, and combinations thereof, were summarized when reported. Preclinical studies were analyzed for mechanistic insights and evidence of superior combination regimens and schedules for potential clinical translation.

## 3. Results

### 3.1. Preclinical Studies

#### 3.1.1. X-Rays vs. Particle Therapies

Preclinical data investigating biological differences between XRT and particle radiation demonstrates significant differences in DNA damage complexity, immune activation, cytokine secretion, and the tumor immune microenvironment (TIME).

##### DNA Damage Response in XRT vs. Particle Radiation

It is well established that high-LET radiation produces more initial and persistent/unrepaired DSBs than XRT at the same physical dose. There is some evidence that high-LET radiation relies more heavily on HR than non-homologous end joining for DSB repair, which is consistent with the notion that high-LET radiation is more complex and difficult to repair DSBs [22,23]. Consistent with this, Suetens et al. compared CIRT (75 MeV/u, LET = 33.7 keV/μm) to XRT in Caco-2 cells and showed more initial and persistent DNA damage at 24 h post-CIRT, as well as more persistent cell cycle arrest after doses of 0.5–2 Gy [24]. Similarly, one study of multiple radiosensitive and radioresistant CRC patient-derived organoids (PDOs) found increased ATM phosphorylation/activation and increased persistent DNA damage foci 24 h post-IR after PBT compared to XRT [25]. Another study found that even small changes in dose-averaged LET (LETd) could alter survival and DNA damage/repair. A comparison of two different LETd levels of PBT (LETd = 2.2 and 7.0 keV/μm; 2 to 4 Gy) in multiple pMMR CRC cell lines found that the higher LETd decreased clonogenic survival and increased persistent unrepaired DNA damage marked by γH2AX foci at 24 h [26]. Since the quality and persistence of DNA damage strongly influence the generation of cytosolic DNA, micronuclei formation, and subsequent innate immune signaling, these radiobiologic differences between XRT and particle therapies are expected to translate into distinct immunologic phenotypes. Several groups have examined whether LET-dependent DNA damage patterns drive differences in immune activation, antigen presentation, and TIME remodeling.

##### Immune Stimulation XRT vs. Particle Radiation

Mechanistic evidence to date on the difference between XRT and high-LET particle therapy in terms of increased immune response with particle therapy differs based on the LET and dose/fractionation of the radiation used, as well as the disease site studied [27]. In CT26 models, XRT and PBT (2–5 keV/μm) were compared at the same dose rate with a regimen of 8 Gy × 3 daily fractions (fx) to primary tumors +/− anti-PD-L1 (10 mg/kg, 3 times per week for 3 weeks, starting on the 1st day of IR) [28]. RNA-seq showed that *Cxcl10* and *Ifnar* increased after both XRT and PBT, but PBT increased TREX1 compared to XRT. This is notable as TREX1 blunts the cGAS-STING (GMP–AMP synthase/stimulator of interferon genes) cytosolic DNA-sensing pathway. While XRT increased CD8^+^ lymphocyte infiltration at 7 days post-IR compared to PBT, notably, the level of CD8^+^ cell exhaustion (as measured by PD1^+^) was lower after PBT. XRT increased natural killer (NK) cells and total PD-L1^+^ cells, while PBT increased the proportion of myeloid-derived suppressor cells (MDSCs) relative to all CD45^+^ cells [28]. A subsequent study on PBT-only (16.4 Gy × 1) showed upregulation of Tlr9 and Irf7, potentially indicating an alternate activation pathway for a Type-1 IFN (T1IFN) response based on endosomal DNA sensing [29]. PBT caused prolonged DNA damage, released damage-associated molecular patterns (DAMPs) (such as ATP, HMGB1, and calreticulin), and increased TAM1, CD8^+^, and CD4^+^ T-cell infiltration. This peaked 7 days post-IR and waned by day 14. In HT29 cells, XRT and PBT (8 Gy) both upregulated PD-L1 and CD47, especially in cells that survived IR, compared to those undergoing apoptosis. This upregulation persisted for at least 6 days post-IR, and for CD47, the degree of upregulation correlated with a poor response to IR as measured by tumor regression grade, although no significant difference was noted between XRT and PBT [30].

Some information can be inferred about the mechanism of immune stimulation, even from studies that did not use direct comparisons between XRT and high-LET radiation. Higher LETd PBT (2–4 Gy, LETd = 2.2 or 7.0 keV/μm) increased micronuclei formation compared to low-LET PBT in vitro at 72 h post-IR [26]. Irradiation of CT26 with 8 Gy PBT at varying LETd (1.09, 2.58, and 7.7 keV/μm) found that the higher LETd PBT increased expression of 41BBL, OX40L, CD40, calreticulin, and MHC-I compared to low LETd [31]. CIRT has also been evaluated in combination with immature dendritic cell (DC) therapy. Although CIRT (LET ~70–80 keV/μm, 2–6 Gy) decreased pulmonary metastases, it did not synergize with immature DC therapy in the Colon-26 model as it did in other disease site models, and there were no direct comparisons with XRT [32].

Overall, these studies highlight the ability of high-LET radiation to enhance cytosolic DNA and T1IFN signaling, promote a more immunogenic TIME, and eventually affect the systemic antitumor immune response with or without ICIs.

#### 3.1.2. Immune Checkpoint Inhibitors (ICIs) with Radiotherapy

Critical issues regarding the optimal clinical combination of IR and ICI in pMMR rectal cancer include whether a specific class of ICI or dual checkpoint inhibition is more favorable than another, whether XRT or high-LET radiation is optimal, the dose/fractionation scheme of the IR, and the optimal timing and sequencing of IR and ICI (e.g., ICI prior to, concurrent with, and/or adjuvant to the IR). These uncertainties call for the need to understand how individual checkpoint pathways interact with radiation-induced innate immune signaling. Preclinical models provide an important window for comparing different classes of ICIs and modalities that will yield the best synergy with IR.

##### Comparing ICI Classes for Optimal Combination Therapy with IR

In CT26 mouse xenograft models, αPD-1 and αPD-L1 administered concurrently with XRT (2 Gy × 5 fx) enhanced both local control and survival. XRT with αPD-1 produced abscopal responses, activated CD8+ T cells, and reduced regulatory T cells (Tregs) and MDSCs [33,34]. Although statistical significance was not reported between XRT+αPD-1 and XRT+αPD-L1, the survival data and tumor rejection in long-term survivors after re-inoculation were roughly equivalent. Similarly, both αCTLA-4 and αPD-1 synergized with XRT (20 Gy × 1 fx) for local tumor control [35].

In a study comparing αCTLA-4 to αPD-1 combined with CIRT in Colon-26 models, αCTLA-4 outperformed αPD-1 with CIRT at both 3 Gy and 10 Gy dose levels in terms of abscopal tumor response, and only αCTLA-4 improved primary tumor control at the 3 Gy CIRT dose level. Interestingly, αPD-1 alone decreased large lung metastases, while αCTLA-4 decreased small lung metastases, and only αCTLA-4 decreased small lung metastases with CIRT compared to CIRT alone [36], with no direct comparison to XRT. CIRT alone (3 or 10 Gy × 1) upregulated PD-L1, CD80, LAG-3, IRF1, and CCL2 expression. One study examined the effects of ICI alone and found that CT26 was responsive to αPD-1 monotherapy, but Colon-26 was not, potentially due to differential Wnt pathway signaling, whereas both were responsive to αCTLA-4 [37]. However, it should be noted that the dose of αPD-1 was much higher than that typically used in other studies in which αPD-1 was combined with IR. Given the heterogeneous responses of αPD-1, αPD-L1, and αCTLA-4 across tumor models and different IR modalities, a more detailed evaluation of immune checkpoint pathways would identify essential advantages of prospective clinical approaches.

##### PD-1/PD-L1 Checkpoint Inhibitors

One study found that delivering αPD-1 prior to XRT slightly increased radioresistance in vitro and increased growth after αPD-1 treatment compared to control in immunocompromised mice in the absence of XRT. However, as both of these experimental settings were immunodeficient, the interpretation of these results becomes difficult [38].

Other combination therapies, including XRT+ICI with agents other than DDRi, may additionally hint at the mechanism behind abscopal responses and the induction of systemic antitumor immunity. Adding cisplatin to XRT (8 Gy × 2 fx) with αPD-1 in C51 cell line models resulted in increased survival and abscopal responses compared to combinations that did not include all three modalities [39]. Bispecific antibodies targeting TGF-β/PD-L1 or PD-1/TGF-β reduced metastases and extended survival in CT26 models by simultaneously blocking immunosuppressive TGF-β and PD-1/PD-L1 signaling [40,41]. XRT with αPD-L1 and PI3Kγi eradicated tumors and increased memory T cells in CT26 models, indicating durable immunity [42]. Adding IL19-IL2 to XRT (5 Gy × 1) and αPD-L1 outperformed XRT + αPD-L1 in CT26, reducing Tregs and boosting CD8^+^ T cells [43], and the TEM1 vaccine with XRT (21 Gy × 1) + αPD-L1 induced abscopal effects in CT26 via vascular targeting [44]. Utilizing irinotecan silicasome + XRT (2 Gy × 4) + αPD-1 produced superior STING and CD8^+^ T cells in CT26 [45]. Advanced delivery systems, including nano metal–organic frameworks (nMOFs) and ROS-responsive nanoplatforms, amplified these effects and achieved complete tumor regression through increased CD8^+^ and IFNγ+ T-cell infiltration [46,47,48]. Adding ES-Cu/galactose-alginate (CSG) hydrogel with XRT (8 Gy × 1) enhanced the abscopal effects by modulating PD-L1 [49]. Even attenuated Salmonella + XRT (5 Gy × 3) + αPD-L1 eradicated tumors via CD8^+^ migration, potentiating ICI+XRT [50].

##### CTLA-4 Checkpoint Inhibitors

Pre-RT administration of αCTLA-4 enhances systemic immunity by depleting Tregs. In CT26 murine models, Young et al. (2016) noted that αCTLA-4 on day 7 before XRT (20 Gy × 1) achieved complete tumor clearance through robust T-cell activation [51]. Crittenden et al. (2018) noted that XRT (20 Gy × 1) with αCTLA-4 in CT26 models required pre-existing CD8+ T-cell immunity to achieve tumor cure, underscoring the need for an established adaptive immune response [35].

Combinations of αCTLA-4, XRT, and other third-generation agents have also been explored. XRT (12 Gy × 1) + IL-12/IL-2/αCTLA-4 induced complete regression in large CT26 tumors (20–40% CR) with abscopal effects and CD8^+^ memory [52]. Chen et al. (2019) combined XRT (8 Gy × 1) with αCTLA-4 and nanoparticles containing catalase and a TLR7 agonist, resulting in primary tumor clearance and abscopal effects, with 60% long-term survival in CT26 models driven by enhanced DC maturation and CD8^+^ T-cell infiltration [53].

##### Dual Checkpoint Inhibition

Dual checkpoint inhibition amplifies the efficacy of XRT by targeting multiple immune regulatory pathways. In CT26 xenografts, combined hypofractionated XRT (8 Gy × 3) with αPD-L1 and αTIGIT achieved complete responses in 90% of cases through enhanced CD8^+^ T-cell activation and granzyme B expression [54]. Additionally, chemoradiotherapy of 8 Gy × 1 fx with cisplatin and 5FU combined with αPD1 and αCTLA4 resulted in 75% complete responses in a CT26 model. This was attributed to a shift in the TIME toward a Th1 phenotype [55].

##### Other ICI Targets/Immunomodulators

Various immune targets have been investigated using IR. ICOS agonists with XRT were found to promote T-cell priming through DC maturation and antigen cross-presentation, thereby improving tumor control [56]. A combination of anti-B7-H3 with XRT (5 Gy × 2–3 fx) and CT26 neoantigen vaccines fully prevented lung metastases by targeting B7-H3-mediated T-cell suppression [57]. XRT (8 Gy × 1) + αCD73 enhanced local and abscopal effects via STING activation, improving tumor control compared to XRT alone [58,59]. A CD47/SIRPα blocking peptide with XRT (8 Gy × 1) was found to be synergistic compared to XRT alone [60]. XRT (2–10 Gy) with αCD40-activated antigen-presenting cells and promoted antitumor effects through DC maturation [61].

Overall, ICIs enhance XRT-induced local and systemic immunity in pMMR CRC models, primarily through CD8+ T-cell activation and pathways, such as cGAS-STING or CXCR3/CXCL10 [62,63]. Direct comparisons between different ICIs and between XRT and high-LET particle therapy are understudied.

##### Optimal Timing and Sequencing of IR and ICI

A critical question regarding the optimal combination of IR and ICIs is the dose/fractionation scheme of IR and the timing of ICI administration relative to IR. Larger doses per fraction of IR in the stereotactic body radiotherapy (SBRT) range (>8 Gy/fx) are thought to be more immune-stimulatory and generate more neoantigens. However, if ICIs recruit radiation-sensitive lymphocytes to the tumor microenvironment, it would be counterproductive to ablate them with large doses of radiation. Preclinical evidence provides some clues as to what may be the most clinically beneficial.

Administration of αPD-L1 synergized with XRT (2 Gy × 5 fx) when delivered on the first and fifth day of XRT, but this effect was completely abolished if αPD-L1 was not delivered until 7 days post-XRT [34]. Lee et al. (2025) compared a single administration of αPD-L1 at 0, 3, 5, and 7 days post-XRT (10 Gy × 1 fx) and found that αPD-L1 administration at 5 days post-XRT maximized tumor growth delay in CT26 models, purportedly by aligning with the peak increase in PD-L1 expression and interrupting an immunosuppressive feedback loop [64]. These divergent results may indicate differences in radiation dose/fractionation schemes. In contrast, αCTLA-4 was more effective when administered 7 days prior to XRT (20 Gy × 1 fx) compared to administration on days 1 or 4 post-XRT [51].

##### Optimal Radiation Dose and Fractionation for Combination with ICI

In preclinical pMMR CRC models, radiation dose and fractionation schedule significantly influence the efficacy of ICI combinations, with ablative and hypofractionated regimes often enhancing potentiation of the immune response.

When αPD-L1 was delivered starting on the first day of XRT in three different dose/fractionation regimens (2 Gy × 18 fx, 8 Gy × 3 fx, and 16.4 Gy × 1 fx), the more conventional fractionated regimen had the best tumor growth delay with αPD-L1 [54]. Boustani et al. compared 2 Gy × 18 fx to 8 Gy × 3 fx (more akin to SBRT) with αPD-L1 starting on the first day of XRT and found that the more conventionally fractionated 2 Gy × 18 fx regimen outperformed the SBRT doses with αPD-L1 either with or without 5-FU [65]. Unfortunately, in both studies, while the radiotherapy regimens themselves were equivalent in terms of theoretically biologically effective doses, they were not equivalent in terms of the observed tumor growth delay, making interpretation difficult.

Conversely, another study demonstrated equivalent tumor growth delays between regimens after XRT alone (3 Gy × 10 fx, 10 Gy × 2 fx, and 15 Gy × 1 fx) in CT26. They found that concurrent αPD-L1 only showed significant synergy with the ablative XRT regimen (15 Gy × 1 fx) and increased CD8+ T-cell infiltration compared with other schedules [66]. Similarly, ablative XRT doses (16 Gy × 1 fx) with concurrent αPD-L1 showed increased antitumor TIME compared to more fractionated regimens (10 Gy × 2 fx and 3 Gy × 10 fx) in CT26 models. While abscopal responses were noted with 16 Gy × 1 fx + αPD-L1, this was not directly compared to other regimens with αPD-L1 [62]. Another study did not find a significant difference between two dosing XRT regimens (7 Gy × 1 fx and 4 Gy × 3 fx), as XRT+αPD-L1 synergized with both compared to XRT alone [33]. One study found that nonhomogeneous dosing strategies (as can be found in SBRT, brachytherapy, or SFRT radiation) may warrant further exploration. In a CT26 model, delivering 16 Gy to 50% of the tumor and 2 Gy to the remainder with concurrent αPD-1 resulted in significant tumor control and enhanced CD8^+^ T-cell infiltration compared with either uniform dosing or 16 Gy to 50% and 0 Gy to 50% of the tumor [67].

Overall, studies with direct comparisons between different ICIs, XRT, and high-LET radiation are lacking. Most studies have shown that ICIs synergize with XRT, while there may be a benefit of αCTLA-4 with CIRT. Synergy with αPD-L1 and XRT appears to be best when administered concurrently or at 5 days post-IR, while αCTLA-4 prior to XRT may be the most beneficial. Data on the optimal dose/fractionation of IR are very mixed for αPD-L1, while dose heterogeneity may be beneficial for αPD-1.

#### 3.1.3. DDRi Radiosensitization

Critical issues regarding the optimal clinical combination of IR+DDRi in pMMR rectal cancer include whether a specific class of DDRi is more favorable than another, whether XRT or high-LET particle radiation is optimal (or has better synergies with specific DDRi classes), and the optimal dose/fractionation scheme of IR for maximal tumor efficacy and minimal toxicity.

##### Radiosensitization of DDRi Combined with Particle Radiation vs. XRT

In other disease sites, PBT (100 MeV, LETd = 9.9 keV/µm) showed more radiosensitization with a variety of DDRi classes compared to XRT [19]. One study found that ATMi radiosensitized high-LET PBT to a greater degree than lower-LET PBT or XRT, which was not the case with ATRi [17]. Similarly, PARPi showed more radiosensitization with higher-LET PBT compared to PBT with lower LET [68]. In pMMR CRC, multiple CRC PDOs were examined in XRT and PBT with multiple different ATMi, and the synergy or lack thereof varied with the specific PDO and ATMi examined [25].

##### Radiosensitization Across Different Classes of DDRi

Sawakuchi et al. compared radiosensitizing agents for clinical translation and studied XRT vs. PBT (100 MeV, LETd = 9.9 keV/µm) in combination with a variety of DDRi (DNA-PKcs, ATM, ATR, PARP, and Rad51) [19]; however, this did not include pMMR CRC cell lines. One study reported similar degrees of radiosensitization with ATMi, ATRi, or PARPi when combined with XRT [69]. Both ATRi and PARPi radiosensitized XRT to similar extents at different dose levels [70].

##### Inhibitors of DNA-PK, ATM, ATR, and PARP

Multiple studies have found that DNA-PK inhibitors (DNA-PKis) significantly radiosensitize pMMR CRC to XRT, but there have been few direct comparisons with other DDRis or high-LET particle therapy [71,72,73]. Another study also assessed XRT+DNA-PKi with 5-fluorouracil (5-FU) and found that the complete response rate increased after DNA-PKi+XRT+5-FU compared to XRT+5-FU alone [74]. ATM inhibitors (ATMis) significantly radiosensitized pMMR CRC cells to XRT [69,75]. In one study, AZD0156 (ATMi) delivered with XRT (5–8 Gy × 2–3 fx) upregulated CXCL10/CXCL11 via STING [76], and another demonstrated that enhancement of MHC-I and IRF1/NLRC5 via NF-κB occurred in a mechanism that was independent of STING [77]. Similarly, PARP inhibitors (PARPis) also radiosensitized CRC cells to XRT [70], with one study finding that XRCC2-deficient cells were particularly radiosensitized [78]. Another study found that PARPi specifically radiosensitized XRT at higher doses per fraction compared to more conventionally fractionated regimens [79]. Similarly, radiosensitization by ATR inhibitors (ATRis) was found to be much more significant in cells lacking functional ATM [80].

##### Other DDRi Classes

CHK1i (e.g., K1586 or preclinical inhibitors) hyperradiosensitizes WRN-deficient CRC by targeting CHK1-p38-MAPK-mediated HRR, increasing DSBs, replication stress, and apoptosis, with strong synergy in p53-mutant lines and in vivo melanoma models [81,82]. TOPKi knockdown reduces DNA damage response proteins (e.g., p-ATM, PARP, and MRE11), increases γH2AX, and sensitizes CRC cells to IR [83]. An inhibitor of XPF-ERCC1 enhanced 5-FU/OXA-based CRT by inhibiting NER, elevating γ-H2AX, and improving tumor control in vitro and in vivo [84].

##### Normal Tissue Toxicity with IR+DDRi

In general, normal tissue toxicity has been understudied in preclinical settings. In preclinical studies of XRT delivered concurrently with DNA-PKi, peposertib was well tolerated in terms of acute weight loss [72,74]. However, both of these studies used flank tumors with shielding, which presumably excluded the abdomen from the field, limiting the extent to which the normal bowel toxicity risk could be surmised. In murine model experiments specifically intended to assess normal tissue toxicity using whole-abdominal radiation fields, ATMi appeared to radiosensitize the normal bowel when using XRT [69]. One study that did not use concurrent DDRi examined differences in normal tissue toxicity between relatively low- and high-LET PBT [26]. By some endpoints, there was more toxicity with high-LET PBT in the small bowel, but there was no significant difference in normal rectal tissue. The same study demonstrated how PBT plans could be generated to place high-LET PBT in gross tumors and away from sensitive normal tissues. This, and the PBT’s lack of an exit dose, could potentially limit the normal tissue toxicity risk of using a concurrent radiosensitizer.

#### 3.1.4. Triple Combination Therapy of IR+DDRi+ICI

##### Efficacy and Mechanisms of Immune Priming with IR+DDRi

Multiple studies have found that triple therapy with high-LET radiation, DDRi, and immunotherapy enhanced antitumor immunity compared to XRT and/or ICI alone, although the proposed mechanisms for how this occurs have differed.

For example, IR can upregulate PD-L1 and CD47, thereby promoting immune evasion. Studies have shown that ATRi mitigates the typical upregulation of PD-L1/CD47 after XRT/PBT, improving both phagocytosis and T-cell priming [30,85]. In CT26 models, ATRi VE822 combined with a single 8 Gy dose of XRT or PBT, along with aPD1 and aSIRPα, resulted in a 60% CR rate, reduced M2 macrophages, and improved abscopal control. PBT/XRT dose-dependently upregulated PD-L1/CD47, which attenuated ATRi and myeloid checkpoint blockade [30]. In HT29 cells, ATRi (VE822) with PBT or XRT (8 Gy) similarly attenuated PD-L1/CD47 upregulation and boosted phagocytosis, with no modality superiority or reported differences in efficacy [30].

One hypothesized mechanism of the synergy of IR + DDRi is the amplification of DNA damage and immunogenic cell death (ICD). Administration of DDRi inhibits DSB repair, amplifies IR-induced damage, and triggers ICD, releasing DAMPs (e.g., HMGB1, ATP, and calreticulin) to activate DC. In CT26 models, ATRi (berzosertib) with XRT (5 Gy × 1) increased γH2AX foci and micronuclei, enhancing ICD and CD8^+^ T-cell infiltration compared to XRT alone [85]. The combination of ATRi + XRT + aPD-L1 produced 88% CRs in CT26 ATM^−^/^−^ (versus 13% WT), with XRT + ATRi activating STING/T1IFN signaling in both the canonical cGAS-STING-pTBK1/IRF3 axis and a non-canonical TRAF6-STING-p65 axis [85].

Similarly, the ATM inhibitor AZD0156 in combination with XRT (8 Gy × 3) plus aPD-L1 achieved 71.4% tumor regression in CT26 cells with favorable tolerability. Triple therapy increased CD8^+^ T cells, reduced Ki-67, and elevated ROS/mitoROS and CXCL10/CXCL11 via STING, outperforming XRT + ATMi and XRT + aPD-L1 [76].

The combination of XRT and ATMi led to both canonical and non-canonical NF-kB activation, leading to MHC-I upregulation. This occurred via IRF1/NLRC5 and was independent of STING. Combining ATMi with XRT or aPD-L1 enhanced pro-inflammatory immune cells in the tumor and their function in the tumor and draining lymph nodes [77].

The PARP inhibitor veliparib plus XRT (8 Gy × 1 fx) increased MHC-I and PD-L1 expression. Adding αPD-1 to fractionated XRT (8 Gy × 2 fx) enhanced tumor regression and survival. The effect was modest in the pMMR cell line CT26 and more significant in the dMMR HCT116 cell line. The effect was abolished by depletion of CD8+ cells, demonstrating a critical component of the systemic antitumor immune mechanism [70].

There is also evidence that the timing and duration of the regimen used to administer combinations of IR + DDRi+ICI may be critical to their efficacy. For example, PARPi (veliparib) with XRT (8 Gy × 2 fx) increased the response to αPD-1, but this effect was ameliorated if the administration of αPD-1 was delayed until 7 days post-XRT [70]. Similarly, when an ATRi (AZD6738) was delivered for only 3 days with 2 Gy × 2 fx of XRT, 4/14 complete responses were obtained, whereas prolonged dosing (9 days) abrogated the benefit [86]. Administration of a short course of ATRi expanded tumor antigen-specific effector CD8^+^ T cells in draining lymph nodes and increased IFN-β/CXCL10 signaling, whereas prolonged ATRi suppressed T-cell expansion.

#### 3.1.5. Diffusing Alpha-Emitters Radiation Therapy (DaRT)

Particle accelerators are among the most expensive medical devices in existence, which limits their access. Radiopharmaceuticals are a promising modality for delivering high-LET radiation (especially when paired with targeting ligands) but lack the degree of control that the external beam enjoys due to dependency on patient physiology and currently suffer from cost and access barriers. DaRT is a promising approach for delivering high-LET alpha particle radiation, which uses seeds coated with Ra-224 that are implanted directly into tumors [87,88], which may help expand access to high-LET radiotherapy. Many implanted alpha sources suffer from short ranges in tissue, which would make them clinically unusable owing to the energy of the alpha particles. DaRT overcomes this issue, as the daughter radionuclides of Ra-224 also emit alpha particles as they diffuse through the tumor tissue. This results in the delivery of a high dose (>10 Gy) over a range of 3–4 mm [88,89,90]. Both the dose heterogeneity of DaRT and the alpha particles themselves may contribute to increased immunogenicity with or without ICIs.

In preclinical studies using the CT26 mouse model, DaRT alone (32.1–50 kBq) reduced tumor growth, induced necrosis, and showed potential systemic immune effects with decreased growth of rechallenge implants after initial DaRT treatment and decreased incidence of lung metastases (93% in controls vs. 56% with DaRT) [87,88,89]. When combined with CpG [88] or Treg/MDSC inhibitors [90], DaRT increased IFN-γ production, reduced Treg and MDSC populations, and promoted a pro-inflammatory TIME with 51% tumor rejection and long-term immunity. DaRT (32.1–33.8 kBq) has also demonstrated synergy with common chemotherapy used in rectal cancer. DaRT + 5-fluorouracil (5-FU) achieved 80% CR, significantly outperforming either treatment alone in CT26 models [89]. In HT29 (pMMR) models, DaRT (75 kBq) with the p53 reactivator APR-246 synergistically enhanced apoptosis via NOXA and cleaved caspase-3 upregulation, attenuating tumor growth in p53-mutant tumors [91].

Unlike traditional low-LET IR, DaRT alpha particles have a short range, causing localized complex DNA damage that is difficult for cancer cells to repair, minimizing harm to healthy tissue. DaRT is a versatile therapy capable of driving both local control and systemic antitumor immunity.

## 4. Discussion

This systematic review synthesizes preclinical evidence supporting the integration of DDRi, ICI, and particle radiation to enhance outcomes in pMMR CRC. Particle radiation induces complex DNA damage and increases cytosolic DNA accumulation, which leads to increased cGAS-STING signaling, T1IFN responses, pro-inflammatory TIME remodeling, and CD8+ T-cell recruitment. This leads to better local and systemic control in immunocompetent murine models. The concurrent use of DDRi and IR has similar effects via increased persistent DNA damage and is a potent radiosensitizer. Combinations of particle radiation or XRT with DDRi and ICI lead to superior tumor regression, abscopal responses, and improved survival. Critical factors include dose and fractionation (hypofractionated XRT or high-LET particle regimens optimized for immune priming) and sequencing, with concurrent or subsequent ICI maximizing efficacy depending on the models and agents used.

Future research should focus on refining the dose and fractionation schedules for IR, as well as the timing of concurrent DDRi and ICI, for maximal efficacy of combination therapy. Additionally, there is a need for more direct comparisons between different classes of IR, DDRi, and ICI to determine the optimal combination to elicit systemic antitumor responses. Although concurrent DDRi use with IR consistently improves local control, questions remain about whether they are necessary when using the more biologically effective high-LET particle radiation for eliciting systemic antitumor immune responses or for local control. Conversely, it is unknown whether high-LET particle radiation is necessary for these objectives or if they can be accomplished with XRT+DDRi for improved local and systemic control with ICI. In general, the normal tissue toxicity of combination therapy is understudied and critical for clinical translation. Further investigation of the influence of various DDR deficiencies (beyond MMR) on therapeutic outcomes will help tailor personalized treatments (Table 1). Identifying additional biomarkers predicting response to specific IR modalities and/or DDRi/ICI classes would also help guide patient selection for early-phase clinical trials.

## 5. Conclusions

High-LET radiotherapy, DDRi, and ICI have synergistic potential for pMMR CRC, with preclinical data demonstrating enhanced ICD, TIME remodeling, and antitumor immunity, leading to superior control and abscopal effects. Prospective integration of these modalities into TNT for locally advanced rectal cancer may improve long-term local and distant control and increase complete response rates for nonoperative management. Combination therapy may be especially advantageous in recurrent and oligometastatic settings where outcomes are poor. Early-phase clinical trials will need to carefully consider normal tissue toxicity risks, especially with concurrent DDRi, and plan radiotherapy fields and normal tissue dose constraints accordingly.

## Figures and Tables

**Table 1:** Potential biomarkers of response to high-LET radiotherapy, DDRi, and ICI.

	Subclass (IR, DDRi, and ICI Class/ Target)	Potential Biomarker/End
Particle/ High-LET IR	CIRT	Cell lines with loss of function of p53 were relatively radioresistant to X-rays but not CIRT [24].
	CIRT	Cells with more intrinsic radioresistance may benefit more from CIRT compared to XRT that intrinsically radiosensitive lines (no specific marker) [92].
	DaRT—alpha	p53 loss-of-function mutations were sensitive to alpha radiation and even more radiosensitized with APR-246 (a p53 reactivating compound) [91].
DDRi	PARPi	XRCC2-deficient cells were especially radiosensitized by olaparib [78].
	ATRi	ATM-deficient cells were more radiosensitized with the addition of ATRi than ATM wild types [80].
	ATMi	Radioresistant CRC patient-derived organoids (PDOs) did not benefit from the addition of 5-FU to XRT but were radiosensitized with ATMi [25].
ICI	αCTLA-4	CT26 models required pre-existing CD8+ T-cell immunity to achieve tumor cures, underscoring the necessity of an established adaptive immune response when treated with XRT (20 Gy × 1) with αCTLA-4 in CT26 models [35].
	αPD-1	One study examined the effects of ICI alone and found that CT26 was responsive to αPD-1 monotherapy, but Colon-26 was not, potentially due to higher Wnt pathway signaling in Colon-26, while both were responsive to αCTLA-4 [37].

Abbreviations: ICI, immune checkpoint inhibitors; DDRi, DNA damage response inhibitors; LET, linear energy transfer; CIRT, carbon-ion radiotherapy; DaRT, diffusing alpha-emitters radiation therapy; IR, ionizing radiation; XRT, X-ray radiotherapy; PARPi, poly (ADP-ribose) polymerase inhibitors; ATRi, ataxia telangiectasia and Rad3-related protein inhibitor; ATMi, ataxia telangiectasia mutated protein inhibitor; αCTLA-4, cytotoxic T-lymphocyte-associated protein 4; αPD-1, anti-programmed cell death protein 1 antibody.

## Data Availability

Data are contained within the article or Supplementary Materials.

## Supplementary Materials

The following supporting information can be downloaded at: https://www.mdpi.com/article/10.3390/cancers18040682/s1, supplementary table.
